# A Combination of Dopamine Genes Predicts Success by Professional Wall Street Traders

**DOI:** 10.1371/journal.pone.0030844

**Published:** 2012-01-24

**Authors:** Steve Sapra, Laura E. Beavin, Paul J. Zak

**Affiliations:** 1 Center for Neuroeconomics Studies, Claremont Graduate University, Claremont, California, United States of America; 2 Department of Economics, Claremont Graduate University, Claremont, California, United States of America; 3 Department of Neurology, Loma Linda University Medical Center, Loma Linda, California, United States of America; Institut Jacques Monod, France

## Abstract

What determines success on Wall Street? This study examined if genes affecting dopamine levels of professional traders were associated with their career tenure. Sixty professional Wall Street traders were genotyped and compared to a control group who did not trade stocks. We found that distinct alleles of the dopamine receptor 4 promoter (DRD4P) and catecholamine-O-methyltransferase (COMT) that affect synaptic dopamine were predominant in traders. These alleles are associated with moderate, rather than very high or very low, levels of synaptic dopamine. The activity of these alleles correlated positively with years spent trading stocks on Wall Street. Differences in personality and trading behavior were also correlated with allelic variants. This evidence suggests there may be a genetic basis for the traits that make one a successful trader.

## Introduction

Financial market volatility is often attributed to institutional traders who are “cowboys,” taking risky positions that result in market paroxysms [1]–[3]. By one estimate, professional traders were responsible for only 10% of New York Stock Exchange (NYSE) trading volume in the 1960's, while individual investors were the primary source of trades. Professional trades are estimated to account for 90% of NYSE activity today, with traders from the 100 largest institutions responsible for 75% of volume [4]. At the same time, stock market prices are known to exhibit “excess volatility” [5], that is, to move more than predicted by the economic fundamentals of companies they represent. The reasons for excessive market volatility are unresolved, but the high volume of institutional trades suggests it is at least partially due to the behavior of professional stock traders [1], [5]–[8] Understanding the behavior of traders is especially important in light of calls for increased regulation of financial markets after the rapid 48% decline in the NYSE in 2008. Recent research has shown that physiologic factors affect financial decisions. For example, Apicella et al. (2008) found that levels of testosterone in saliva were associated with greater risk-taking in a laboratory investment game [9]. Field studies have also highlighted this relationship, demonstrating that when professional traders trade in volatile markets they have increased cardiovascular tone [10], and that morning testosterone levels predict professional traders' daily profits [11]. Both prenatal and circulating testosterone appear to influence the choice of a financial career and success in this field as measured by longevity [12], [13].

The biological factors affecting financial decisions may have a genetic basis. Analyzing data from Swedish twins, 29 percent of the variation in the decision to invest in stocks can be attributed to genetics [14]. Data from twins who are investment professionals showed that approximately 25 percent of portfolio risk is due to one's genes [15]. This research is tantalizing because it points towards genes affecting financial behaviors; yet it does not indicate the genes that cause differences between individuals. Our approach was to fill this gap by identifying specific genes in individuals who are making significant financial decisions on a daily basis. Understanding how biology affects financial decisions may have policy implications as the U.S. grapples with additional regulation of financial institutions.

In the present study we genotyped 60 New York City professional stock traders (those who execute trades in stock markets) at five different companies during the summer of 2008. All samples were obtained before the prodigious stock market decline that began August 28, 2008 that destroyed several large money management companies. Studying Wall Street professionals can be difficult because of the lavish value of their time and their high turnover, especially among short-tenure traders. In this study, we targeted traders who were successful; that is, those who on average have survived the ups and downs of Wall Street for nearly a decade. The majority of the traders assessed were intermediary traders who bought and sold securities for clients rather than on behalf of the companies for whom they worked.

Rather than measure personality traits of traders [16] or study college students making low-stakes decisions in a laboratory [17], we investigated whether a set of functional genetic polymorphisms associated with brain function would predispose one to be successful on Wall Street. In particular, we were interested in determining whether frequencies of certain alleles would differ between traders and controls. We investigated two regions in the dopamine receptor 4 gene, one region in the catecholamine-O-methyltransferase gene (COMT), a region in the monoamine oxidase A gene (MAO-A), and the promoter region in the serotonin gene (SERT). These genes were selected because of their relationship to risk-taking and mood. Dopamine in particular has been associated with financial decision-making in functional MRI (fMRI) studies of financial decisions [18], [19]. Genes map more directly onto the traits that may make traders successful than do brain imaging studies that characterize the state of brain activity during a particular task. Yet, findings from neuroeconomics studies [20] coupled with methods for reducing the genomic search space in genetic association studies guided our choices of candidate genes. Successful traders have to balance risk-return trade-offs throughout the day. At the same time, stock traders have to execute trading rules while maintaining discretion when unforeseen opportunities present themselves. This requires modulating attention, novelty seeking, learning, and impulsivity. We hypothesized that sustaining the balance between rules and discretion would be associated with a particular combination of genes and alleles that affect brain function.

Levels of synaptic dopamine have an inverted-U relationship with several cognitive abilities as shown by both animal and human data [21]. For example, markedly low or high levels of dopamine are associated with disruptions in working memory, while moderate levels of dopamine are associated with improved memory [22]. Densities of D2 and D3 dopamine receptors also have an inverted-U association with sensation seeking [23] and risk-taking can be increased by administration of the dopamine agonist pramipexole [24]. Evidence from Parkinson's disease patients also shows an association between treatment with dopamine agonists and risk-taking and impulse control disorders [25], [26]. Adolescents, who have fewer dopamine receptors and reduced synaptic dopamine compared to adults, show elevated risk-taking compared to pre- or post-adolescents [27]–[29]. Since successful trading depends on appropriate amounts of risk-taking and engaging cognitive control, we hypothesized that successful traders would have genes that produce intermediate levels of synaptic dopamine.

## Materials and Methods

### Participants

A total of 60 traders from five Wall Street firms and 54 graduate students from the Claremont Graduate University in Southern California participated in the study. The trader population consisted of 54 males and six females. The anonymity of participants was maintained throughout, with the data we collected in New York City in July, 2008. The companies who agreed to make their employees available to us traded high volumes of equities, fixed income instruments, and derivatives for both clients and their own proprietary trading books. The controls consisted of 44 males and 10 females who were students at Claremont Graduate University, producing a total sample size of 114. These data were collected in September, 2008 from the lobby of the Claremont Graduate University's Peter F. Drucker-Masatoshi Ito Graduate School of Management over several evenings when MBA courses were ending. We targeted MBA students in order to recruit controls who were interested in business, but were not professional traders. All participants provided written informed consent, and the study protocol was approved by the Institutional Review Board at Claremont Graduate University. Saliva samples were collected from all participants using the Oragene® DNA kit (DNA Genotek Inc, Ontario, Canada) and genomic DNA was purified according to manufacturer's specifications. The concentration and purity of each DNA sample was determined using a Nanovue spectrophotometer (GE LifeSciences, Piscataway, NJ, USA).

### Genetic Targets

Our approach was to look for a set of genetic alleles active in the brain that may affect success in finance. As mentioned above, we focused on genes that affect the activity of the neurotransmitter dopamine (DA), because a large number of neuroeconomics studies have demonstrated that DA influences financial decision-making. Functional MRI (fMRI) studies have associated activation in dopamine-receptor-rich ventral striatal regions in the brain with the anticipation of financial gains [19], [30]. These studies indicate DA is associated with financial decision-making.

Two recent studies of financial decisions in the laboratory have associated variations in the dopamine receptor 4 (DRD4) gene with greater risk-taking by college students [31], [32]. DRD4, a G-protein coupled receptor located on chromosome 11, contains two distinct repeat polymorphisms designated the promoter insertion/deletion (DRD4P) and the exon III (DRD4e3) variable number of tandem repeats (VNTR) [33]. College-aged participants who had 7 or more VNTRs in DRD4e3 region took more risk than those with less than 7 repeats. The DRD4P region contains two alleles, a long allele (L) and a short allele (S), with the length of the allele inversely associated with DRD4 transcription levels [34]. Alleles associated with a decrease in DRD4 activity in the prefrontal cortex have been associated with novelty seeking, attention deficit hyperactivity disorder, and substance abuse [35]–[37]. The effect of DRD4P on financial decisions has not been previously reported.

Genetic variation in genes altering dopamine activity may also influence financial decision-making. The COMT gene is involved in the catabolism of dopamine in the frontal cortex and the ventral striatum. This gene contains a coding region single nucleotide polymorphism (SNP) within exon 4 in which codon 158 may be either a valine (Val) or a methionine (Met) [38]. The methionine variant has lower enzymatic activity than the valine version resulting in higher synaptic DA. Individuals who are homozygous for the Met allele (denoted A/A) exhibit only 25% of the enzymatic activity compared to individuals homozygous for the Val allele (G/G) [38]. The low activity Met allele has been associated with reduced levels of phasic dopamine in subcortical regions [39]. Several studies have found that G/G individuals perform better than those with the A/A allele on tasks demanding cognitive flexibility, while individuals with the A/A allele are better at tasks demanding focused attention [40]-[43]. Additionally, A/A individuals show higher loss aversion behavior, decreased impulsivity and reduced novelty seeking [44]–[46].

Monoamine oxidase (MAO) is also involved in the catabolism of dopamine, and allelic variants of the MAO-A gene have been associated with sympathetic arousal and anxiety that may affect financial decision making [47]–[49]. Two recent studies investigated financial decisions and MAO-A variants. The 3 VNTR allele has low enzymatic activity that produces higher levels of synaptic DA. Conversely, the 4 VNTR variant has higher activity, resulting in lower levels of DA. The low DA (4 VNTR) allele has been associated with a preference for long-shot risks and to a lower likelihood of obtaining protective insurance [50]. Another study reported that those with the high DA (3 VNTR) MAO-A allele had an increased likelihood of taking an advantageous financial risk [51].

Serotonin (5-HT) is well known for its effect on anxiety and mood [52]-[54]. The promoter region of the serotonin gene, called 5HTTLPR or SERT, has two alleles, short (S) and (L) with serotonin activity higher for the L variant. The S allele is associated with greater activity in the amygdala, a brain region often associated with vigilance and fear [55]. SERT has been associated with risk preference, with those homozygous for the short version of the allele (S) exhibiting 28% less risk-taking than those with S/L or L/L variants [32]. Those homozygous for the S allele show greater loss aversion and take fewer financial risks [56], [57]. Using a similar methodology, a recent study found no relationship between SERT alleles and risk propensity [51].

### Genotyping

Genotyping was performed by PCR as previously described [58]. A subset of the genotypes was independently verified by DNA sequencing of the PCR products at UC Davis Sequencing Core (Davis, CA). Genotyping for the COMT Val158Met (G/A) SNP was performed by tetra-ARMS PCR as described in Ruiz-Sanz, Aurrekoetxea, del Agua, & Ruiz-Larrea (2007) [59]. Genotyping for the DRD4 exon III VNTR and MAO-A promoter VNTR was performed at the UCLA Genotyping Core (Los Angeles, CA). The genotyping failure rates were 1% for the DRD4 exon III VNTR, 1.8% for the MAO-A promoter VNTR, 1% for the DRD4P, and 2.6% for the COMT G/A SNP.

### 2.4 Statistical Analysis

Genotype frequencies for the two biallelic polymorphisms (DRD4P and COMT Val158Met SNP) were tested for Hardy-Weinberg Equilibrium (HWE) in the controls using both chi-squared and Fisher's exact test (both results were consistent, only chi-squared is reported). Genotype frequencies for the multiallellic DRD4 exon III VNTR were tested for Hardy-Weinberg Equilibrium in the control group using the Markov-chain algorithm [60]. The MAO-A promoter VNTR is an X-linked, multiallelic polymorphism; therefore, testing for Hardy-Weinberg Equilibrium was performed only in the female controls as described in Philibert, Gunter, Beach, Brody, & Madan (2008) [61]. The genotype frequencies of all the polymorphisms were in Hardy-Weinberg Equilibrium in the control sample (DRD4 VNT: *p* = 0.47; DRD4P: *p* = .21; COMT: *p* = 0.16; MAO-A: *p* = 0.62). Distributions of the genotypes also matched those described previously for similar samples [31], [32], [51], [58], [62], [63].

Testing for association in traders and controls with the DRD4 promoter insertion/deletion, and COMT G/A (Val158Met) SNP was performed using logistic regression and assuming a dominant genetic model for the DRD4 l (long) and COMT A (Met) alleles, respectively, as described in Balding (2006) [64]. Testing for association with the SERT promoter VNTR was performed by excluding individuals with 1 or more copies of the rare SERT alleles as described above, and treating the polymorphism as biallelic in the remaining population (54 traders and 52 controls). Association was tested using logistic regression and assuming a dominant genetic model for the s (short) allele also as described in Balding (2006) [64]. We tested for association with the X-linked MAO-A promoter VNTR by first excluding heterozygous females and performing the analysis on the remaining subjects, males and homozygous females as described in Fossella et al. (2002) [65], using a codominant genetic model for the 3R and 4R alleles (3 and 4 repeat alleles respectively). Association with the multiallelic DRD4 exon III VNTR was performed by classifying genotypes by the number of copies of the 7R allele (7 repeat) and subsequently using logistic regression assuming a dominant genetic model for the 7R allele as described in Kuhnen and Knutson (2005) [17]. We additionally tested for association by grouping the 2R and 7R alleles, as there is biochemical evidence these alleles confer diminished biological activity to the DRD4 protein [66]. We subsequently classified genotypes according to the number of copies of the 2R or 7R alleles for logistic regression analysis. Neither approach showed a statistically significant association. Confidence intervals (CI) were computed using logistic regression. All tests for association were performed using a significance level of *p*<0.05.

Dopamine activity assignments were based on research cited above. These were COMT: GG = 0 GA = 1 AA = 2; DRD4P: LL = 0, LS = 1, SS = 2. The DA index simply summed each of these values to create an overall DA index. We developed this approach as a simple and direct measure to assess the combination of genes on behavior.

## Results

The average age of the trader sample was 35.4 years (SD 7.3; range = 22–51) and the average number of years of professional trading experience was 9.2 (SD 7.0; range = 1–26). The average years of experience did not vary by firm. The average age of the control sample was 32.4 years (SD = 10.6; range = 22-69), and this did not differ significantly from the age of the trader sample (*p* = .09). Removing the 69 year old participant does not change the results so this participant was not excluded.

### Genetic analysis

The DRD4e3 2R, 4R and 7R VNTRs were observed at the highest frequencies (>10%), with other alleles (3R, 5R, 8R and 10R) occurring at frequencies < 10% in the traders and controls. We classified subjects as carriers or non-carriers of the 7R allele as done in previous studies [31], [32]. Twenty-nine percent of traders had the 7R allele, while 24 percent of controls carried this VNTR. This difference was not significant (chi-squared test, *p* = 0.61).

For DRD4P, we classified subjects as carriers or non-carriers of the S allele. Since the S allele is associated with increased expression of DRD4 mRNA [34], S carriers are expected to have higher levels of DRD4 mRNA and higher receptor levels than non-carriers (L/L individuals). We found that more traders were homozygous for the L allele compared to controls (70% vs. 53%) and that traders were nominally less likely to be S carriers than controls (30% vs. 47%; chi-squared test, *p* = 0.06).

**Figure 1:**
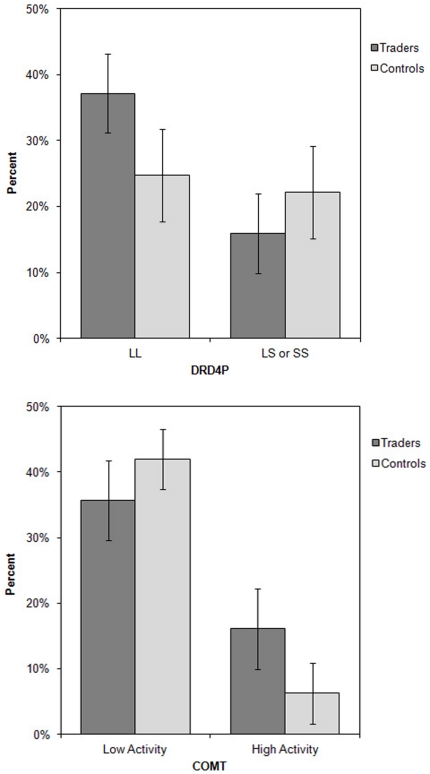
Professional traders were 36% less likely to carry the high activity S allele of the DRD4P gene than were controls (Professionals: 30%, Controls: 47%; P = **.06).** Traders had the high activity A/A COMT allele 138% more than controls compared to the lower activity A/G or G/G alleles (Professionals: 31% A/A; Controls: 13% A/A; P = .019).

For the COMT SNPs, we categorized those with A/A as having higher synaptic dopamine (H) than those in the G/G or A/G lower COMT activity (L) group. Traders were significantly more predominate in the H group (31%) relative to controls (13%) (chi-squared test, *p* = 0.019; Fig. 1).

We also tested whether variants of the MAO-A gene were more predominant in traders. We found no significant difference between traders and controls for the 3 VNTR versus 4 VNTR alleles (chi-squared test, *p* = 0.44). In addition, there was no difference between traders and controls for the L and S alleles of the serotonin promoter region, SERT (chi-squared test *p* = .90).

Next, we examined whether combinations of genetic polymorphisms might be more frequent in traders relative to controls. We found a statistically significant COMT X DRD4P interaction (F test, *p* = 0.014). This interaction shows that traders are more likely to have the H COMT allele and have at least one S allele for DRD4P. No other combinations of polymorphisms resulted in a significant interaction.

### Years working on Wall Street

**Figure 2:**
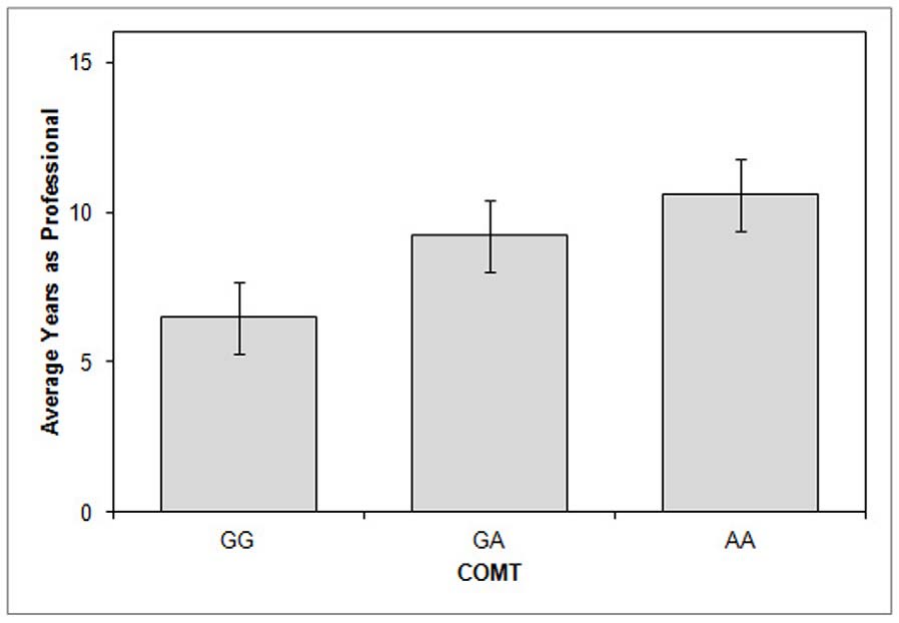
Years working as a trader correlates with DA levels as determined by COMT alleles (r = **.19>0, one-tailed t-test P** = **.05).** Those with the lowest COMT-related synaptic DA category (G/G) averaged 6.5 years of trading experience, while those in the highest category (A/A) averaged 10.6 years trading stocks (one tailed t-test P = .05).

It is difficult to gauge an individual trader's success based on portfolio performance because returns depend on overall activity in the stock market, growth of the economy, as well as the quality of their company's research. We therefore used years working as a measure of success as a trader since low performing traders are routinely laid off and typically move into different professions. Although this definition may not encompass all aspects of success, it provides a consistent measure of performance and has previously been used [12]. To assess the relationship between genetic polymorphisms and years working on Wall Street, we assigned values to each allele for synaptic DA activity (see Methods). We then correlated these indices with years working as a professional trader. COMT associated DA levels had a positive and significant correlation with success as a trader (*r* = .19, one-tailed t-test, *p* = .05); Fig. 2. Neither DRD4P (one-tailed t-test, *p* = .93) nor DRD4e3 (one-tailed t-test, *p* = .95) were associated with years trading.

To investigate the relationship between DRD4P, COMT, and success on Wall Street more fully, we constructed a linear index based on the amount of synaptic dopamine each allelic combination would produce (see Methods). Performing and 80–20 split, we found that that those with 15 or more years on Wall Street had a DA index 35% higher than those who had spent less time trading professionally (DA index long tenure mean = 1.80, short tenure mean = 1.33; F = 3.02, *p* = .04; Fig. 2). We also found that those with the highest scores on the DRD4P index were less likely to trade in volatile markets, based on self report, than traders with lower DA levels (*r* = −.30, one tailed t-test, *p* = .01).

### Personality traits

A subset of 25 traders completed the Myers-Briggs Type Indicator (MBTI) to measure their personality traits. We included this analysis to map the genotype of successful traders into a phenotype. There was no difference between those who completed the MBTI and those who did not for gender, age, or years traded (t-tests, *p*>.20), indicating that this subsample is representative of the larger sample of traders. The MBTI is a standard test of personality based on four dichotomous categories that correlate with the “big five” personality groupings [67]. We were specifically interested in the distribution of the fourth category in the MBTI, Judging (J) or Perceiving (P), as this dimension is thought to represent how much organization one prefers in life. Those who are the J type typically carefully plan their lives, while those who are the P type tend to keep their options open [68]. In the trader sample, 44% had the Judging (J) personality type and 56% were the Perceiving (P) type. We expected that more successful traders would be type P, as individuals with this personality type tend to do significant analysis before making decisions. The P personality type in the U.S. population is estimated to be 46% [69], though the trader sample is not significantly different than this value (binomial test, *p* = .21). We found that the P personality type had a 50% higher score on the DA index than did traders of the J type (mean J = 1.09; mean P = 1.64; one-tailed t-test, *p* = .036). We did not find differences for any of the other MBTI types.

Genetics also affected outlook on the world. Traders with the high activity COMT allele and those who scored higher on the overall DA index were less likely to view the world in terms of “survival of the fittest” (COMT index: *r* = −.38, two-tailed t-test, *p* = .004; DA index: *r* = −.30, two tailed t-test *p* = .027).

## Discussion

Our primary finding is that two genetic alleles that affect DA are associated with success at trading stocks on Wall Street. We showed that alleles of COMT and DRD4P were predominant in traders compared to controls, and we also demonstrated that a combination of these alleles were associated with being a trader. Both COMT and DRD4P affect the brain's synaptic levels of dopamine. Several neuroeconomics studies of financial decisions have shown that activity in the dopaminergic nucleus accumbens predicts risk-taking [18]. It is possible that the successful traders in our sample who had genes associated with moderate synaptic dopamine may be predisposed to take an appropriate level of risk, but not too much, thereby contributing to their success. This was evidenced by their reduced reports of trading in volatile markets.

Using years on Wall Street as a measure of success, we found a positive correlation between success as a trader and a combination of alleles that are associated with intermediate levels of synaptic DA. We did not find that traders were more likely to carry the 7R variant of the DRD4e3 VNTR, an allele associated with risk-taking in two laboratory studies. Our results suggest that successful traders in our sample weigh risk and reward, rather than taking excessive risks. This was born out in the personalities of our sample of traders. We found that they were good at integrating disparate pieces of information, eschewed trading in volatile markets, and did not view the world as threatening their survival. These findings align with previous evidence suggesting that more experienced traders may respond in a less emotional way than those with less experience [10]. Nevertheless, other studies of professional traders have found that they are susceptible to trading biases [70]–[72].

We also examined two other genetic targets that affect DA levels and personality traits: MAO-A and SERT. We found no significant difference in the frequency of MAO-A or SERT alleles in traders compared to controls. This is potentially problematic, given that other studies have linked SERT and MAO-A to financial risk-taking tendencies; however, these results have been inconsistent [51], [56], [57].

It is important to note that our observations come from a relatively small sample. High income individuals recruited at work, such as the Wall Street traders, are difficult to access for data collection, which is the reason for the small sample at hand. It is therefore important that the results reported here are replicated. Additionally, our sample of traders may not include some of the most successful professionals who have the opportunity to leave the types of major companies from which we drew our data for early retirement or to begin their own company. We opted to define successful trading by longevity because this information could be reliably collected and because this is a measure also used in previous research [12]; however, other definitions of success may be considered more informative and should be examined in the future.

Our findings can contribute to discussions of changes in the regulation of finance professionals and asset markets in the wake of the 2008 recession. Our results suggest that using a history of risk-taking and competitive behaviors when hiring traders could be a mistake, though this is often done [73]. Having too little or too much risk-aversion is not associated with success by those in our sample; rather taking a balanced level of risk appears to be optimal. Further, our findings indicate the importance of training the dopamine system to accurately assess risk and reward in the context of trading. The balancing of risk and reward is essential to successful trading, but trading on another's behalf may skew the way risk is assessed [74]. Recent research from our lab has shown that without training the dopamine system, losses are accentuated even when trading for one's own account [75]. Lastly, our findings indicate that hiring more female traders may improve investment company returns. Nonprofessional female investors tend to be more risk-averse, trade less, and earn higher returns [76]–[78].

So, what makes a professional trader successful? Combining the personality analyses and genetic findings from the present study, reveals that our sample of traders are analytical, integrative, and can delay gratification. They have a genetic profile associated with balanced levels of dopamine, and also linked to moderate but not high risk-taking behavior. Thus, successful traders do not appear to take extraordinary risks and also appear to take a longer-term perspective. Our analyses indicate that these traits may have a genetic predisposition.
